# Reevaluating Electroencephalography Monitoring in Koolen-de Vries Syndrome: A Case of Delayed Focal Impaired Consciousness Seizure Diagnosis

**DOI:** 10.7759/cureus.83693

**Published:** 2025-05-07

**Authors:** Ryan Nazari, Manav Nayeni, Pavan Sakhamuru, Anuj Gupta, Charles V Moylan

**Affiliations:** 1 College of Osteopathic Medicine, Kansas City University, Kansas City, USA; 2 Department of Pediatrics, Pediatric Associates – Children’s Mercy Health Network, Kansas City, USA

**Keywords:** eeg, epilepsy, fics, focal impaired awareness seizures, focal impaired consciousness seizure, focal seizures, kansl1, kdvs, koolen-de vries syndrome, pediatric seizure

## Abstract

Koolen-de Vries syndrome (KdVS) is a rare multisystem genetic disorder due to deletions in the KANSL1 gene. The most common type of seizure documented in these patients is focal impaired consciousness seizure (FICS). These seizures present with difficult-to-distinguish characteristics, including autonomic symptoms, brief loss of consciousness, and post-ictal confusion. The ambiguity of this presentation can make it difficult to detect clinically. This case presents an eight-year-old male child with KdVS who presented to the clinic with complaints of increased outbursts, spatial disorientation, issues with mood and self-regulation, and episodes of “spacing out” as noted by his teacher. The initial diagnosis was dysautonomia and was conservatively managed. However, due to worsening neurocognitive outcomes, neurological referral and work-up were initiated to further elucidate the etiology of his symptoms. The patient's electroencephalography (EEG) findings showed frequent focal sharp waves consistent with FICS. He was then treated accordingly with diazepam and amantadine, which led to a significant improvement in neurological status. This case highlights the importance of the use of EEG in KdVS patients, as well as the implications of implementing guidelines recommending the low threshold required for use of EEG and full neurological work-up for patients with any alarm symptoms possibly indicative of FICS or other epileptiform activity.

## Introduction

Koolen-de Vries syndrome (KdVS) is a rare, multisystem, neurodevelopmental disorder caused by microdeletions on chromosome 17q21.31 or by a truncating variant in the KAT8 regulatory NSL complex unit 1 (KANSL1) gene [1-2]. KdVS is characterized by congenital malformations, developmental delay, intellectual disability, childhood hypotonia, epilepsy, dysmorphisms, and behavioral features [1-4]. As genetic testing continues to advance and become more readily available, phenotypic heterogeneity within the syndrome is becoming more evident, with increased insight into the roles of specific genetic variants on clinical phenotypes [2]. Some level of psychomotor developmental delay is noted in nearly all individuals with KdVS from an early age, with the majority of individuals functioning in the mild-to-moderate range of intellectual disability [1-3].

Neurologically, epilepsy is a significant concern for patients with KdVS. Epilepsy, including generalized seizures and unilateral clonic seizures, is noted in approximately 33% of affected individuals [1]. The most frequent seizure type in these patients is focal impaired consciousness seizures (FICS), observed in up to 65% of cases with seizure activity [5]. These focal seizures often manifest with non-motor or autonomic features such as verbal unresponsiveness, lack of awareness of the ongoing seizure, automatisms, post-ictal confusion, and increased frequent focal sharp wave activity on electroencephalography (EEG), all of which are symptoms that individually can be misinterpreted as behavioral or psychiatric issues [5-6]. Particularly in pediatric patients, symptoms such as inattention or irritability associated with confusion can be attributed to attention-deficit disorders, autism spectrum behaviors, or cognitive disability, potentially delaying appropriate neurological evaluation and targeted treatment [2,4-5,7-8]. This underscores the importance of thorough neurological evaluation in patients with KdVS presenting with such symptoms to ensure timely and appropriate management.

Despite the high prevalence of epilepsy and the known risk for occult seizure activity in KdVS, there are currently no standardized guidelines for routine or serial EEG monitoring as an adjunct in this population [1]. Diagnostic practices remain largely symptom-driven; thus, the known associations to specific neurocognitive processes like FICS and other seizure types in KdVS can lead to missing these sequelae if supportive evidence, like EEG, to confirm a clinical diagnosis is absent. These processes can present with subtle and easily misattributed symptoms, leading to a lack of comprehensive workup and attribution to behavioral or developmental comorbidities. This diagnostic ambiguity is especially problematic in pediatric KdVS patients, where unrecognized early epileptiform activity may contribute to long-term neurocognitive impairment [5,9]. As such, a more proactive approach to neurological surveillance in KdVS may be warranted. We present a case in which a pediatric patient diagnosed with KdVS shortly after birth presented to the clinic multiple times with autonomic and attention-related complaints and was not given a full neurological work-up until years later. This resulted in a subsequent diagnosis of FICS, and proper treatment led to a significant reduction in neurocognitive symptoms.

## Case presentation

An eight-year-old male child presented to the pediatric clinic with his parents due to a recent history of behavioral changes in the form of new-onset social outbursts and “spacing out” episodes noted by his teachers. His medical history consisted of KdVS, confirmed genetically at birth. Microanalysis testing at the time of diagnosis demonstrated an interstitial deletion of at least 661 kb within the cytogenetic band 17q21.31, encompassing the KANSL1 gene and an extended deletion of the LRRC37A4P gene. He demonstrated typical phenotypic features associated with KdVS at birth, including dysmorphic facial features, hypotonia, and intellectual disability. His musculoskeletal features included bilateral hypoplastic 12th ribs and mild scoliosis, findings that are rarer when coinciding with KdVS, and may represent an expanded phenotype related to the extended deletion of the LRRC37A4P gene. 

The patient’s parents noted that at around five years of age, the patient was experiencing social outbursts at school, “spacing out” episodes, and amnesia related to routines that were previously well-known. He was also experiencing forgetfulness related to the location of familiar items in his house, which was determined to be episodes of spatial disorientation. These symptoms prompted his parents to seek a medical evaluation. Limited testing at the time was inconclusive, and the patient’s symptoms were attributed to dysautonomia. The patient did not undergo EEG testing at the time. Subsequently, his symptoms were conservatively managed.

**Figure 1:**
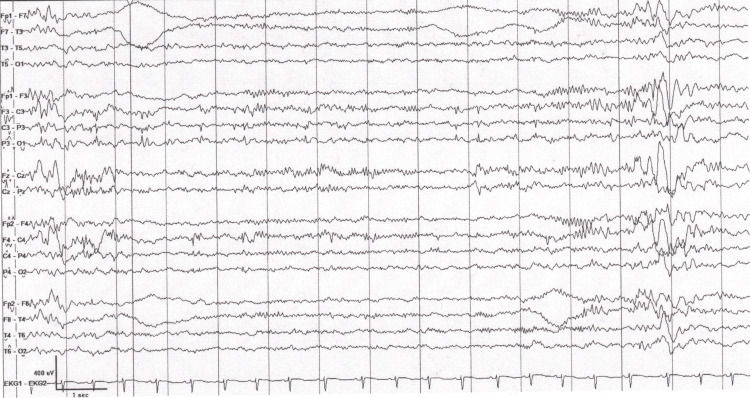
EEG done upon neurology referral. There are frequent sleep-augmented multifocal sharp wave discharges in the left temporal, parietal, and right central head regions, suggesting focal cortical irritability with a lowered seizure threshold. The averaged spike-wave index was 26%, not meeting criteria for CSWS. These findings are seen in patients with focal onset seizures. EEG: electroencephalography; CSWS: continuous spikes and waves during sleep

At the time of presentation to the pediatric clinic, the patient reportedly experienced further worsening of his condition despite conservative management, and was experiencing more frequent episodes of “spacing out” as noted by his teachers. His mother also noted that following one of these episodes, the patient had vomited significantly. Due to her concern, the patient was brought into the clinic to initiate a neurology referral. Physical examination at the time was non-contributory, with no generalized or focal neurological signs noted. Review of systems was non-contributory apart from the self-reported “spacing out” episodes with concurrent vomiting as well as spatial disorientation. The patient was subsequently referred to neurology to further elucidate the etiology of his symptoms. An EEG demonstrated epileptiform abnormalities consistent with seizure activity. Notably, his EEG showed frequent focal sharp wave activity in the left temporal, parietal, and right central head regions, which did not meet definitive criteria for continuous spikes and waves during sleep (CSWS). EEG findings in conjunction with clinical work-up led to a diagnosis of FICS. The EEG can be seen in Figure 1.

Based on the neurological findings and clinical discretion, the neurologist elected to trial the patient on daily oral diazepam therapy, despite not meeting criteria for CSWS, to assess for potential therapeutic benefit. In addition, daily amantadine was initiated off-label as an adjunct for cognitive stimulation. The combination of these two medications substantially improved the patient’s neurocognitive functioning. In particular, the patient’s school performance gradually improved. However, the patient’s mother eventually noted that his therapeutic regimen triggered problematic sedation. He was then cautiously tapered off the diazepam to reduce his sedation. The concern at the time was that his neurocognitive function may decline with the benzodiazepine taper, however, his subsequent clinical neurologic evaluations unexpectedly demonstrated persistent improvement. The use of amantadine was also discontinued at the same time. He showed significant improvement post-treatment for his seizure activity, notably in attention, memory, and academic performance. These improvements illustrate that his possibly undiagnosed seizure activity as early as five years of age may have contributed to his history of neurodevelopmental impairment. He remains relatively symptom-free to this day and continues to be progressing well academically and socially.

## Discussion

This case presents a unique perspective on the management of KdVS patients in the pediatric setting. In particular, the focus on clinical indications for performing EEGs for KdVS patients presenting with possible FICS symptoms is highlighted within this case. Literature is plentiful regarding the possibility of FICS being the most common type of epileptical pathology in KdVS patients, especially with concurrent autonomic features such as syncope, palpitation, and epigastric sensations [1,5,10]. A summary of some common features of KdVS noted in a 2023 study, which collected self-reported data from KdVS patients, can be found in Table 1. Given these autonomic features, it is possible that these symptoms can have a significant impact on the daily functioning of patients living with FICS in relation to their KdVS. The autonomic features of FICS have a compounding effect on KdVS patients, where the stress and unpredictability of the seizures can worsen anxiety and social withdrawal [1]. The challenges associated with accurately diagnosing FICS in KdVS can lead to overall worsening of neurocognitive and psychosocial factors in terms of long-term prognosis [1,11]. Thus, it is imperative that FICS and its associated autonomic features be promptly identified, worked up through testing, and treated to prevent worsening of long-term outcomes in a disease that otherwise has a favorable prognosis [1,5,7,10-11]. This clinical picture was evident in our patient, as once his FICS was identified and appropriately pharmacologically treated, his neurocognitive measures improved greatly. 

**Table 1:** Frequency of certain clinical features noted in a 2023 study of 237 KdVS patients with the KANSL1 gene mutation The information presented in this table is derived from the GenIDA Patient Registry study for KdVS [8]. Not all 237 participants responded to each question in the questionnaire from the study of origin [8]. KdVS: Koolen-de Vries syndrome

General Features	% cases (n)
Males	48.9 (116/237)
Speech or language delay	73.6 (128/174)
Neurological features	
Epilepsy	47.5 (97/204)
Behavioral problems	54.8 (109/199)
Hypotonia	61.5 (139/226)
Skeletal features	
Scoliosis	25.5 (51/200)
Joint laxity	50.0 (100/200)

The autonomic features and “spacing out” episodes described in KdVS patients experiencing FICS can often be misattributed to other causes, as was the case in our patient, whose initial symptoms were correlated to dysautonomia without further workup. This misattribution led to a delay in our patient’s epilepsy testing and subsequent management. Pellinen et al. described how delays in diagnosing focal non-motor seizures can lead to increased morbidity [12]. Additionally, it is imperative to identify epilepsy in the setting of KdVS to prevent the risk of prolonged seizures [1]. Diagnostic challenges are also worsened by the fact that autonomic features are often the sole presenting epilepsy-specific symptoms [1]. Staring spells, described as “spacing out” episodes in our patient, are often attributed to other psychiatric conditions in the pediatric setting, such as attention-deficit hyperactivity disorder [13]. It is clear that there is a possibility of misdiagnosing epileptic pathology in KdVS patients, as was seen in our patient. It may be beneficial to provide education guided towards the recognition of FICS and staring spells to parents of pediatric KdVS patients. 

There currently do not exist particular guidelines for the scheduled monitoring of epileptic pathologies in KdVS patients. Current literature suggests that the frequency of EEG monitoring in KdVS should be based on specific clinical indications rather than a fixed schedule or other symptoms [1,5]. These indications specifically include new neurological findings or changes in muscle tone [1]. The utility of EEG testing, especially for identifying focal sharp wave changes, cannot be overstated [5]. As of late, neurology guidelines recommend continuous EEG monitoring only in critically ill patients [14]. In critically ill children, prolonged EEG monitoring only marginally improves epileptiform change detection capabilities at higher costs [15]. Most cost-benefit analyses of serial EEG monitoring exist in the setting of critically ill children. No literature exists that explores serial EEG monitoring at any intervals specifically for KdVS patients. Given the previous discussion of how neuropsychiatric symptoms of FICS, such as dysautonomia and staring spells, are often misattributed to other conditions, there exists some scope to explore the concepts of interval and continuous EEG monitoring for clinically ambiguous symptoms in KdVS. Our patient saw significant neurocognitive improvements after the correct identification and management of his epileptiform pathology. Had he gotten a full neurological workup, including an EEG due to suspicion of possible epileptic activity at his earlier visits, there may have been the opportunity to prevent the interval deterioration of his neurocognitive and psychosocial outcomes. Although it may not be possible to do serial EEG monitoring in an asymptomatic KdVS patient due to clinical and financial limitations, there may be some utility in having a low threshold in resorting to ambulatory EEG systems, such as wearable EEG monitoring devices, at the first onset of any dysautonomic or other neuropsychiatric symptoms. These tools can capture abnormal EEG activity not readily identified on an in-clinic EEG.

A limitation of this case is the absence of explicit documentation regarding the EEG montage used; however, in an epilepsy monitoring unit, it is standard practice to review multiple montages, including the average montage, during interpretation. Despite this, the clinical correlation and findings strongly support the diagnosis presented. Additionally, it is important to note that the patient's episodes were classified as FIC seizures, in accordance with the updated nomenclature in the International League Against Epilepsy (ILAE) guidelines released in April 2025, which replaced the term 'focal impaired awareness seizures' (FIAS) [16].

Nonetheless, we implore that further research should explore the cost-benefits of doing such interval serial EEG monitoring and the implications of maintaining a low threshold for ambulatory EEG monitoring versus current guidelines of testing, which recommend testing based solely on clinical indications, for the early detection of seizure activity in KdVS patients. Early and effective seizure identification and control enhance overall quality of life [17], which is of the utmost importance in pediatric patients with KdVS.

## Conclusions

This case report highlights the importance of early intervention in preventing neurocognitive and psychosocial decline in pediatric patients with KdVS. Our patient's outcomes suggest that at least a portion of his cognitive regression was likely due to undiagnosed FICS secondary to KdVS. This regression could have been prevented if diagnosis and treatment had been initiated up to three years earlier at the time of initial symptom presentation.

Given the high prevalence of epilepsy in KdVS, relying solely on clinical symptoms to initiate neurological work-up can delay diagnosis and necessary treatment. We propose for consideration utilizing a low threshold indication to initiate a full neurologic work-up for suspected epileptic activity, including possible use of EEG to support the clinical diagnosis. To further improve development and quality of life in KdVS patients, future studies should explore the overall effectiveness of interval serial or continuous EEG monitoring with more sensitive guidelines versus clinical indication-only testing.
